# Quantifying effects of blood pressure control on neuroimaging utilization in a large multi-institutional healthcare population

**DOI:** 10.1371/journal.pone.0298685

**Published:** 2024-04-30

**Authors:** Theodore R. Welch, Aliza Yaqub, Danny Aiti, Luciano M. Prevedello, Zarar A. Ajam, Xuan V. Nguyen

**Affiliations:** 1 Department of Radiology, The Ohio State University College of Medicine, Columbus, Ohio, United States of America; 2 Bahria University Medical and Dental College, Karachi, Pakistan; 3 Canton Medical Education Foundation, Canton, Ohio, United States of America; 4 The Ohio State University, Columbus, Ohio, United States of America; Faculty of Medicine, Saint-Joseph University, LEBANON

## Abstract

**Objectives:**

Essential hypertension is a common chronic condition that can exacerbate or complicate various neurological diseases that may necessitate neuroimaging. Given growing medical imaging costs and the need to understand relationships between population blood pressure control and neuroimaging utilization, we seek to quantify the relationship between maximum blood pressure recorded in a given year and same-year utilization of neuroimaging CT or MR in a large healthcare population.

**Methods:**

A retrospective population-based cohort study was performed by extracting aggregate data from a multi-institutional dataset of patient encounters from 2016, 2018, and 2020 using an informatics platform (Cosmos) consisting of de-duplicated data from over 140 academic and non-academic health systems, comprising over 137 million unique patients. A population-based sample of all patients with recorded blood pressures of at least 50 mmHg DBP or 90 mmHg SBP were included. Cohorts were identified based on maximum annual SBP and DBP meeting or exceeding pre-defined thresholds. For each cohort, we assessed neuroimaging CT and MR utilization, defined as the percentage of patients undergoing ≥1 neuroimaging exam of interest in the same calendar year.

**Results:**

The multi-institutional population consisted of >38 million patients for the most recent calendar year analyzed, with overall utilization of 3.8–5.1% for CT and 1.5–2.0% for MR across the study period. Neuroimaging utilization increased substantially with increasing annual maximum BP. Even a modest BP increase to 140 mmHg systolic or 90 mmHg diastolic is associated with 3-4-fold increases in MR and 5-7-fold increases in CT same-year imaging compared to BP values below 120 mmHg / 80 mmHg.

**Conclusion:**

Higher annual maximum recorded blood pressure is associated with higher same-year neuroimaging CT and MR utilization rates. These observations are relevant to public health efforts on hypertension management to mitigate costs associated with growing imaging utilization.

## Introduction

Hypertension is a common chronic disease with high morbidity and mortality. According to CDC statistics, the prevalence of hypertension in adults over 20 years of age in the United States is 50% [1]. Moreover, optimal blood pressure control in the hypertensive population is difficult to achieve, with a third of patients in the Antihypertensive and Lipid Lowering Treatment to Prevent Heart Attack Trial failing to achieve the goal of blood pressure of <140/90 mmHg at 5-year follow-up [2]. Hypertension is a major cardiovascular risk factor due to the relationship between high blood pressure, stroke and cardiovascular mortality [3]. Since the brain is susceptible to early effects of hypertension-induced organ damage [4], numerous neurological disease process are potentiated or exacerbated by poorly controlled hypertension [5]. A prior meta-analysis had shown that in middle-aged adult patients, each 20 mmHg increase in SBP doubles the stroke death rate, with more pronounced increases in mortality risk in older subjects, without any significant threshold effect [6].

Despite the relatively asymptomatic course of essential hypertension prior to end-organ injury, the healthcare costs attributable to hypertension approach tens of billions of dollars annually [7]. Given the increasing prevalence of hypertension in the general population over the past several decades [8], it is plausible that downstream utilization of imaging exams for hypertension-associated diseases such as stroke will increase, posing challenges to national efforts to control healthcare costs. One subset of healthcare expenditures that has been targeted for cost reduction is medical imaging, particularly relatively high-cost imaging modalities such as CT and MR. For instance, MRI accounts for over half of all diagnostic expenditures in outpatient neurology nationally [9]. However, the relationship between hypertension control and radiologic imaging has not been studied extensively. Except at certain extremes, such as symptomatic hypertensive emergency, hypertension by itself is usually asymptomatic and typically not an indication for radiologic imaging, so the impact of hypertension on imaging is likely complex and multifactorial, driven both by incidence of associated diseases like cerebrovascular disease or by the need to exclude them. The latter can vary across practices and settings, since in one study, an estimated 22–28% of radiologic imaging examinations are ordered due to perceived risk of litigation [10], whereas in another study, only 1% of the variation in emergent imaging utilization was attributable to physician preference or discretion as opposed to patient and visit-level factors [11].

Given the need to better characterize the impact of hypertension on neuroimaging utilization at the population level, we seek in the current study to quantify the relationship between maximum blood pressure recorded in a given year and utilization of neuroimaging CT or MR in the same year. This study seeks to employ large-scale multi-institutional data available in existing electronic medical records to quantify the empirical relationship between maximum annual blood pressure and same-year neuroimaging in a large multi-institutional dataset. We hypothesize that increasing blood pressure disproportionately increases both CT and MR neuroimaging utilization.

## Methods

### Data sources and queries

This retrospective cohort study was determined to be exempt from human subjects research review by the Office of Responsible Research Practices at the Ohio State University, and patient consent was not obtained, as the authors do not have access to information that could identify individual participants during or after data collection. Data were obtained using Cosmos (Epic Systems, Verona, WI), an informatics-based application that provides a data-mining framework to aggregate electronic health data for research [12]. This HIPAA limited data set, at the time of this writing, consists of more than 137 million patients from 142 different organizations. We used the Population data model within this application to obtain aggregate numbers of patients with blood pressure (BP) data documented under Vitals for diastolic blood pressure (DBP) and systolic blood pressure (SBP). The data for this study were accessed on 04/01/2022, 11/14/2022, and 01/02/2024.

For each of the 2016, 2018, and 2020 calendar years, queries were performed to retrospectively identify the number of patients with blood pressure recorded in that calendar year that is equal to or greater than pre-defined threshold values [50 (minimum threshold), 80, 90, 100, 110, and 120 mmHg for DBP and 90 (minimum threshold), 120, 140, 160, 180, and 200 mmHg for SBP]. In addition, at each threshold value, the subset of patients who also had a neuroimaging exam of interest in the same calendar year were also counted. The rationale for quantifying same-year imaging utilization for a BP cohort is that it would help answer the question of how BP affects likelihood of imaging over a defined interval, so as to capture the aggregate effects of all downstream diagnoses or conditions for which the BP cohort is at risk over a 1-year period. Also, by using a 1-year interval, we were able to avoid effects of seasonality on imaging utilization. For the purposes of this study, the neuroimaging exams counted were any brain/head MRI or CT exams and any CT /MR angiographic studies of the head, neck, or both. For instance, the study includes neck CTA or MRA exams but not soft tissue neck or cervical spine CTs or MRs. MR and CT utilization was recorded separately. Note that utilization in this analysis is treated as a binary patient variable, such that the counting method does not differentiate between a patient undergoing multiple neuroimaging exams and a patient undergoing only one such exam.

For the most recent calendar year studied, an additional analysis was performed in different age groups to assess the potential impact of age as a confounder. The data collection process described above was repeated for each of 4 age categories (0–24, 35–44, 45–64, and ≥65 years). Similar analyses were performed for subsets of the cohorts defined by payor type (Medicare, Medicaid, Self-Pay, Private/Other) and for selected encounter types (Emergency, Inpatient Admission, Surgery). In this way, potential confounding effects of age, encounter type, and payor status on the BP-utilization relationship are minimized. Similarly, the most recent calendar year of the study was also used to analyze the potential effects of cerebrovascular accident (CVA) diagnoses on the BP-utilization relationships. For each BP cohort, the prevalence of a same-year billing diagnosis of CVA was computed, and the MR and CT utilization rates were computed for this subset of each BP cohort in a similar manner as described in the preceding paragraph.

In addition, to provide a general assessment of health status of these BP cohorts, the same-year all-cause mortality rates of each cohort were computed, with age adjustment performed by weighting age-specific mortality based on the relative age composition of the entire population within the Cosmos dataset at the time of analysis.

### Data analysis

From the aggregate counts described above, the numbers of patients in specified ranges of annual maximum blood pressure and the counts of the subset of these patients undergoing neuroimaging were calculated. For each blood pressure range, the ratio of these numbers yielded the proportion of patients undergoing neuroimaging in the same calendar year. Data are presented for each calendar year and for each range of maximum annual blood pressure values as percentages of patients undergoing CT or MR neuroimaging in the same calendar year, with diastolic and systolic BPs analyzed independently. For the most recent calendar year studied, 95% confidence intervals were computed for each data point, based on the normal approximation of the binomial. Inferential statistical analyses for the relevant imaging utilization comparisons were performed using the chi-square test.

Note that this study was not designed as a regression analysis for individual-level prediction but rather as a cross-sectional observation of “all-cause” imaging, analogous to epidemiological studies of “all-cause” mortality for a population of interest. Rather than attempting to identify and analyze all potential diagnoses and conditions that could impact imaging utilization, our approach in this study was to succinctly capture aggregate effects of all possible downstream diagnoses and clinical indications affecting likelihood of imaging into a single probability of “all-cause” imaging for a BP cohort.

## Results

The Cosmos analysis included over 38 million patients. The number of patients included, along with the subsets undergoing CT or MR utilization are shown in Table 1. Multi-institutional Cosmos data encompassing a broad mix of institutions had overall utilization for CT neuroimaging of 3.8–5.1% and MR neuroimaging of 1.5–2.0%. Mild increases in utilization over time were observed, with an absolute change of 0.5% for MR and 1.4% for CT over the calendar years analyzed, corresponding to a relative increase of 33% for MR and 37% for CT. Interval changes in both CT and MR utilization were statistically significant at p<0.00001.

**Table 1:** Total patients examined in the local institutional dataset in the multi-institutional Cosmos analysis^a^.

Calendar Year	Total N	Total CT	%	Total MR	%
2020	38,361,412	1,971,294	5.1%	761,001	2.0%
2018	36,833,714	1,682,988	4.6%	671,891	1.8%
2016	21,764,928	819,446	3.8%	323,584	1.5%

^a^Counts shown are based on available DBP values.

**Fig 1:**
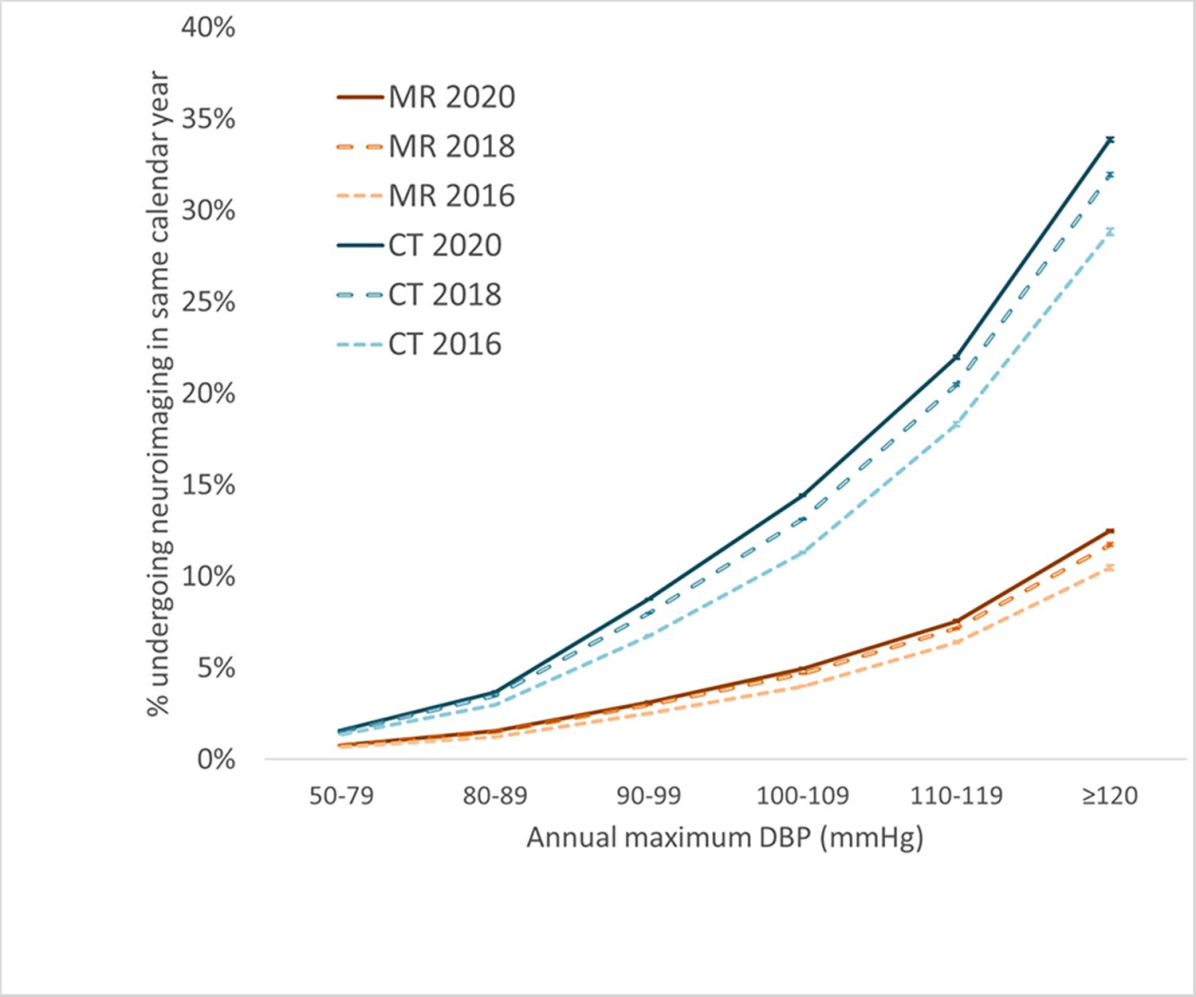
Neuroimaging utilization increases with increasing annual maximum DBP based on multi-institutional data accessed via Cosmos. Error bars denote 95% confidence intervals based on the normal approximation to the binomial.

**Fig 2:**
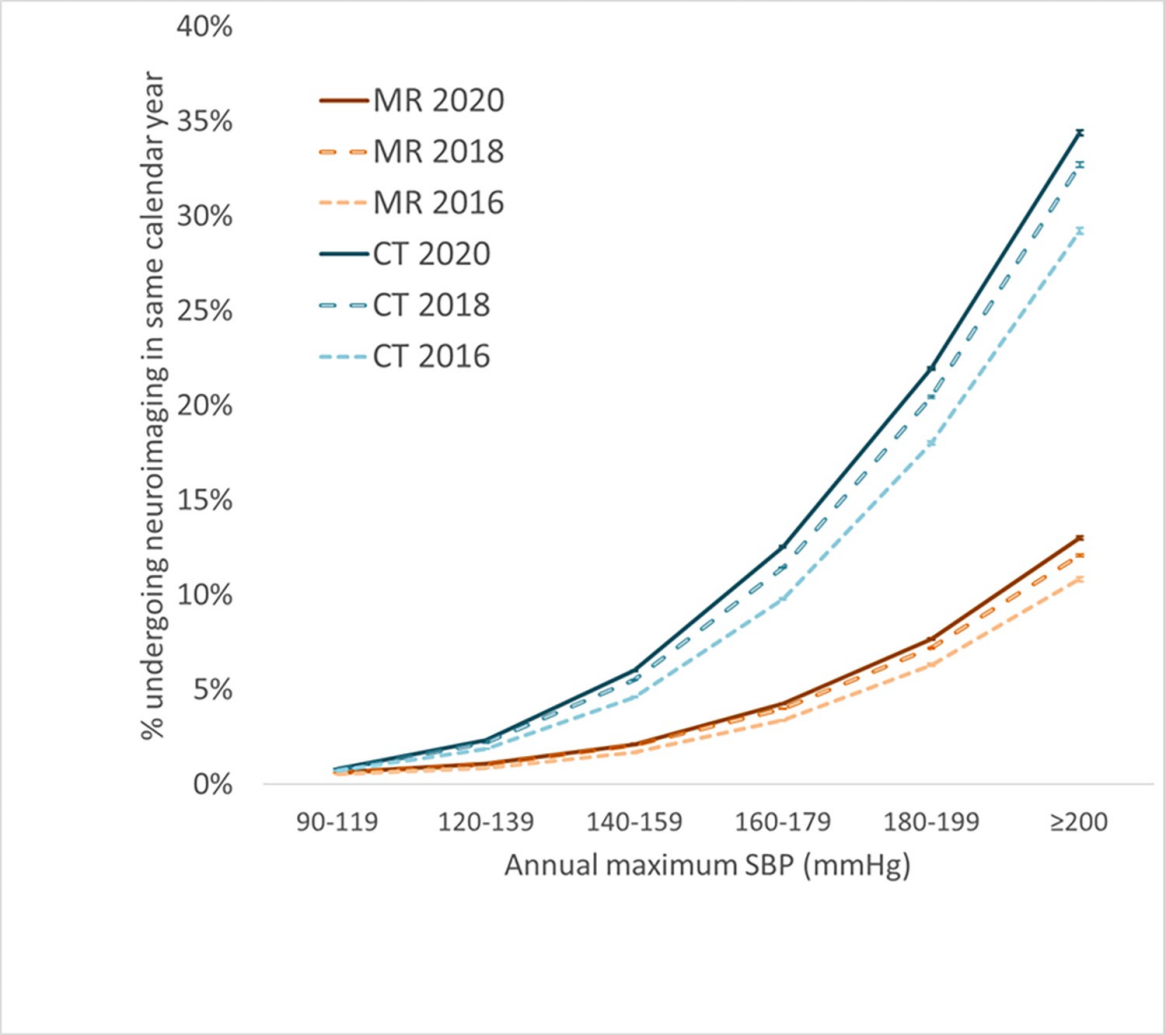
Neuroimaging utilization increases with increasing annual maximum SBP based on multi-institutional data accessed via Cosmos. Error bars denote 95% confidence intervals based on the normal approximation to the binomial.

Figs 1 and 2 shows the relationship between neuroimaging utilization and maximum annual blood pressure. In the multi-institutional Cosmos population, utilization increases monotonically with annual maximum BP, without an apparent threshold effect. There is a steep dependence of utilization rates on BP, with changes of as much as 20-fold and 40-fold in MR and CT utilization, respectively, across the range of BPs examined. Note that the relationship of utilization to blood pressure is not linear, with disproportionately higher frequency of neuroimaging at higher blood pressure ranges. Even a modest increase in BP to 140 mmHg systolic or 90 mmHg diastolic is associated with 3–4 fold increase of MR utilization and 5-7-fold increase in CT utilization in the same calendar year compared to BP values below 120 mmHg / 80 mmHg. For each year of analysis, the CT and MR utilization rates for BP ranges above the lowest examined range were statistically significantly higher than for the lowest BP range at p<0.00001.

**Fig 3:**
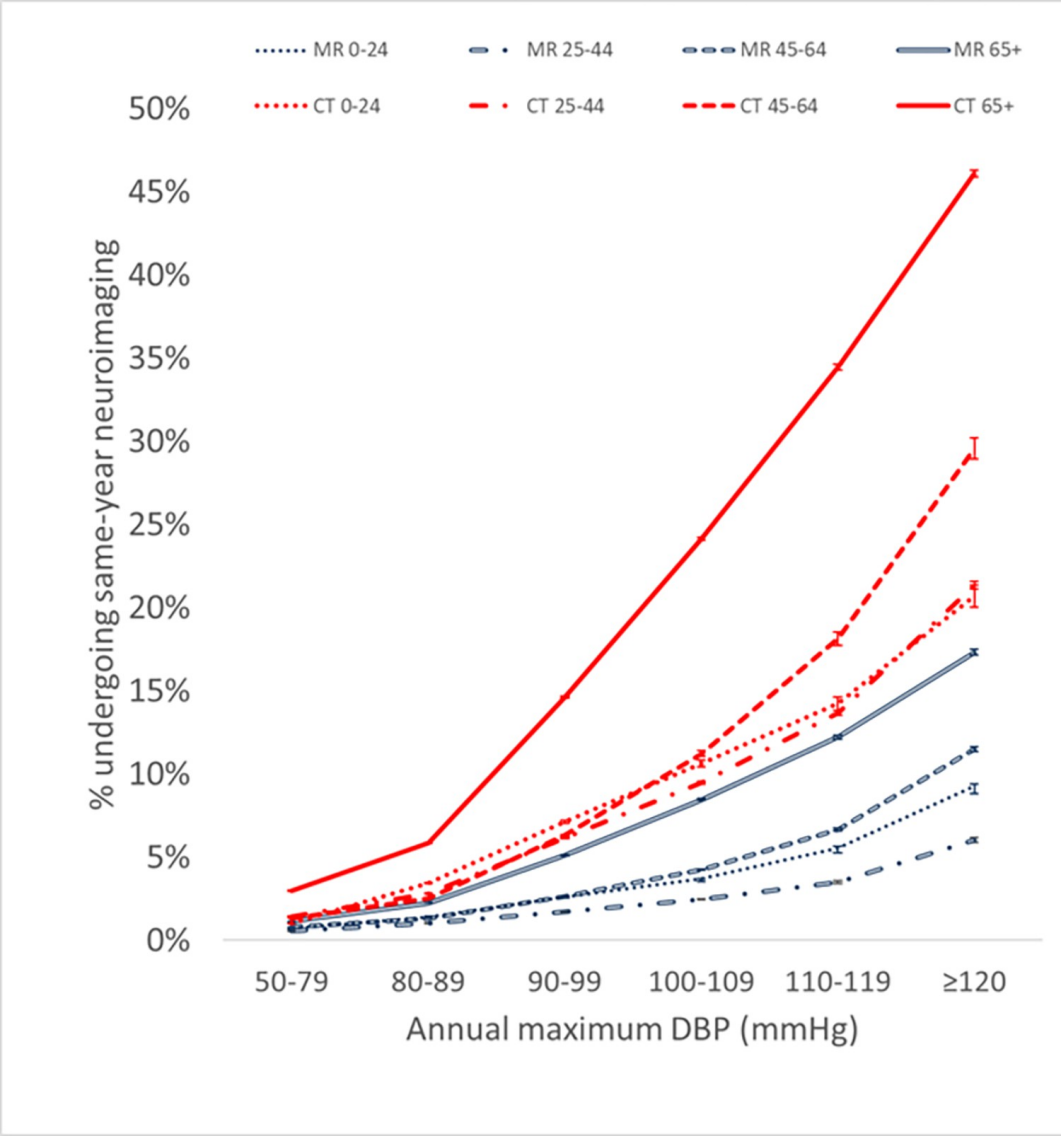
Same-year neuroimaging utilization within various age ranges increases with increasing annual maximum DBP based on multi-institutional data accessed via Cosmos. Error bars denote 95% confidence intervals based on the normal approximation to the binomial.

**Fig 4:**
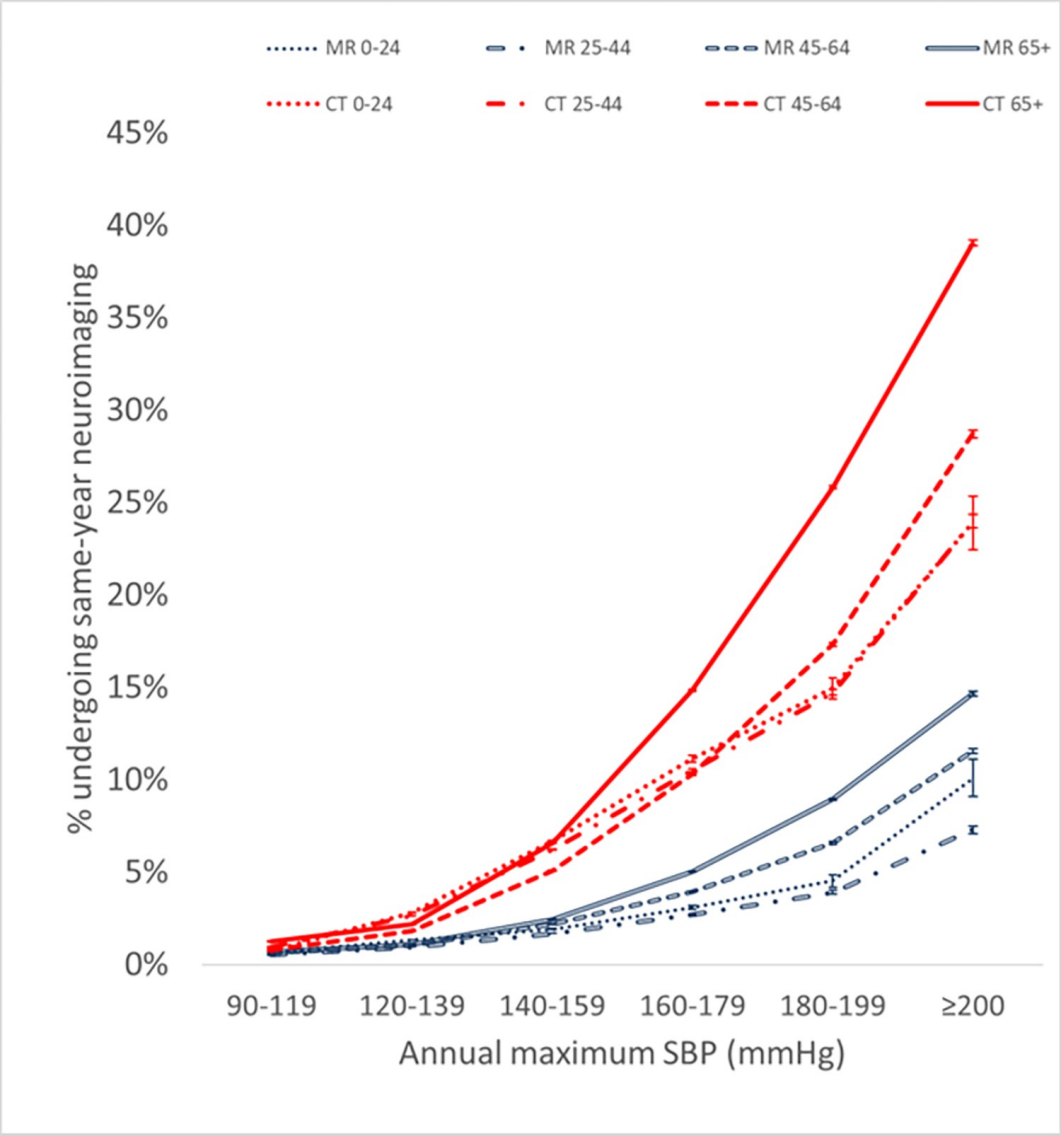
Same-year neuroimaging utilization within various age ranges increases with increasing annual maximum SBP based on multi-institutional data accessed via Cosmos. Error bars denote 95% confidence intervals based on the normal approximation to the binomial.

To assess whether a confounding effect of age may explain the observed relationship between BP and neuroimaging utilization, each population was partitioned into 4 age cohorts for the most recent calendar year examined. A similar positive nonlinear relationship between utilization and BP persists across all age groups, but slopes and average values of the utilization-BP curves are higher in older patients (Figs 3 and 4). Compared to the lowest BP range, the CT and MR utilization rates for the other BP ranges are statistically significantly higher at p<0.00001 for each age group.

Since CVA is a diagnosis for which elevated blood pressure is a risk factor, we attempted to ascertain how much of the observed BP-utilization relationships could potentially by explained by CVA diagnoses. As shown in Table 2, a same-year billing diagnosis of CVA was found in 2% of the lowest-risk BP cohort, in contrast to over 10% of the highest-risk BP cohort. There is a positive correlation between BP and MR/CT utilization within the CVA-positive subset of the population (Table 2), but magnitude of the BP effect is smaller than for the whole study population due to relatively high utilization rates in the lowest-risk CVA-positive BP group, which is not unexpected given the common practice of neuroimaging during workup and follow-up of stroke presentations regardless of blood pressure.

**Table 2:** Prevalence of same-year CVA billing diagnoses and rates of MR and CT neuroimaging within the CVA-positive subset of each BP cohort.

DBP [mmHg]	Less than 80	80–90	90–100	100–110	110–120	120 or more
Prevalence of CVA	2.4%	3.0%	4.0%	5.6%	7.7%	10.3%
MR utilization among CVA patients	28.5%	32.4%	39.8%	46.2%	50.1%	51.0%
CT utilization among CVA patients	42.4%	48.3%	60.7%	71.1%	77.7%	80.4%
SBP [mmHg]	Less than 120	120–140	140–160	160–180	180–200	200 or more
Prevalence of CVA	2.4%	2.8%	4.2%	6.7%	9.8%	13.0%
MR utilization among CVA patients	32.2%	29.3%	32.1%	37.0%	42.4%	47.4%
CT utilization among CVA patients	48.8%	43.4%	49.1%	58.0%	66.4%	73.4%

**Fig 5:**
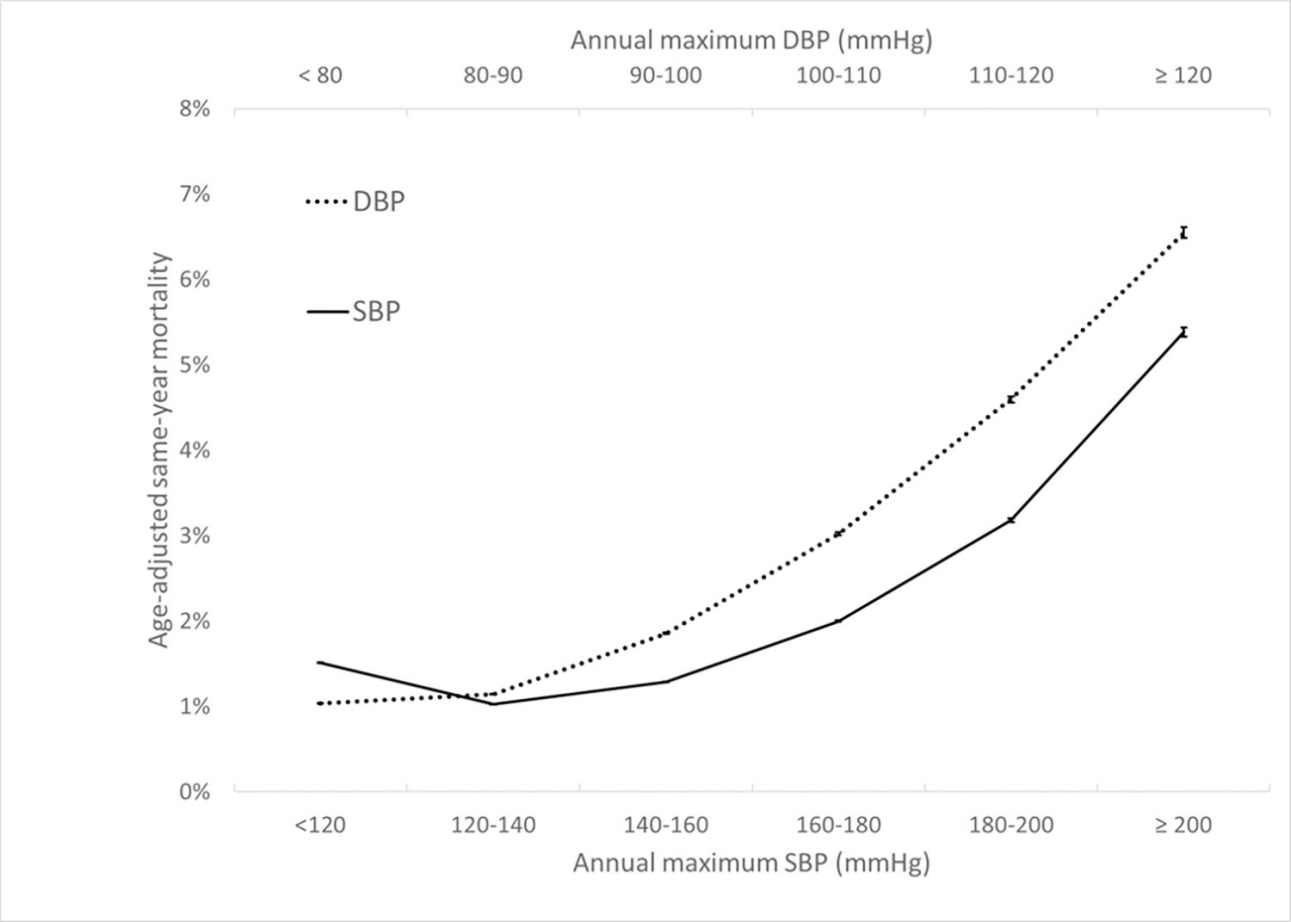
Age-adjusted same-year mortality rates for the DBP and SBP cohorts. Error bars denote 95% confidence intervals based on the normal approximation to the binomial.

To facilitate interpretation of the observed imaging utilization rates relative to the health status of these BP cohorts, rates of same-year mortality were computed for the most recent calendar year of the study period. As shown in Fig 5, the age-adjusted mortality for each BP cohort generally increases as maximum annual BP increases, with the highest-risk BP cohorts showing 5–6 times higher rates of same-year mortality than for the lowest-risk BP cohorts.

**Fig 6:**
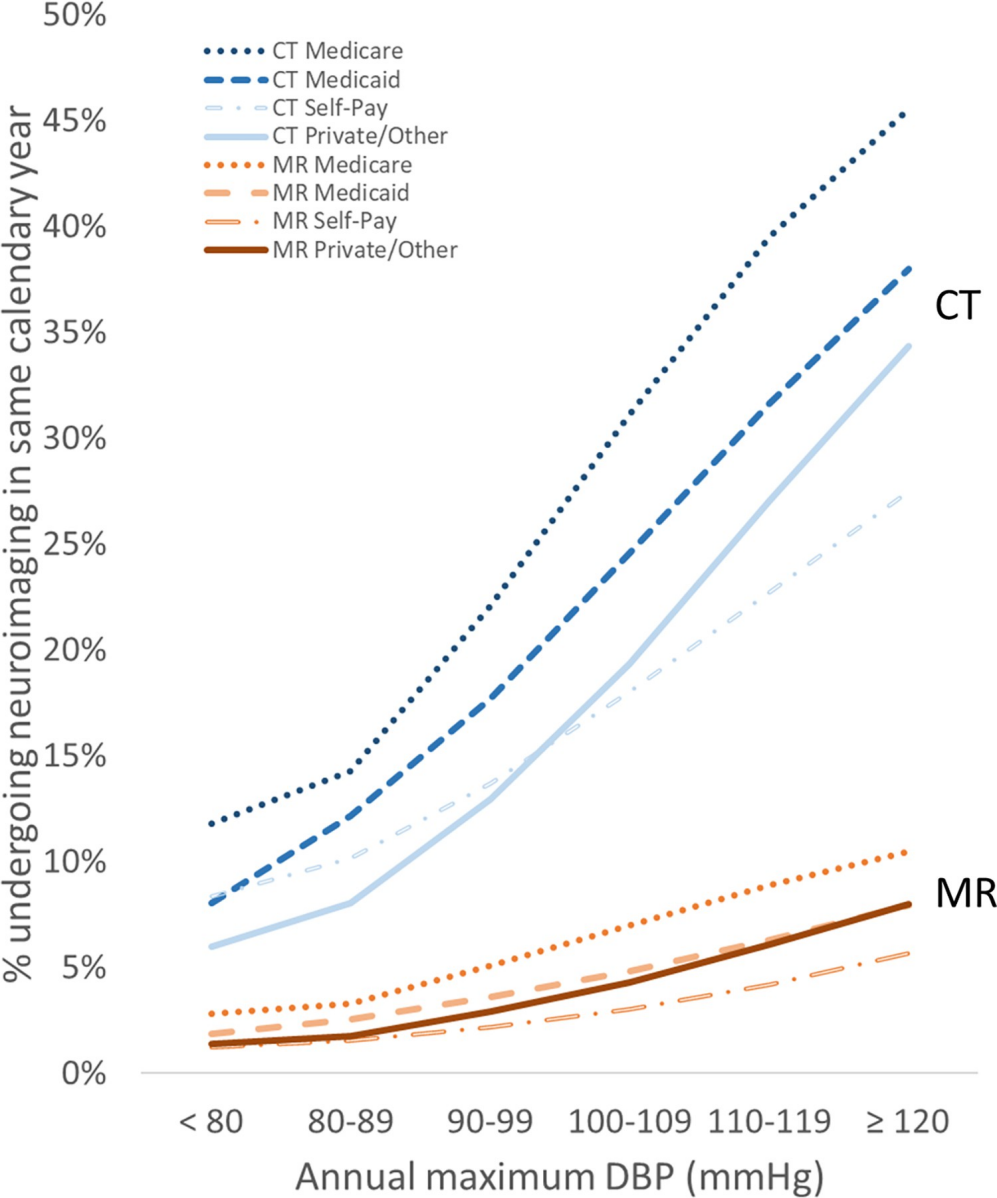
Same-year neuroimaging utilization stratified by payor type increases with increasing annual maximum DBP.

**Fig 7:**
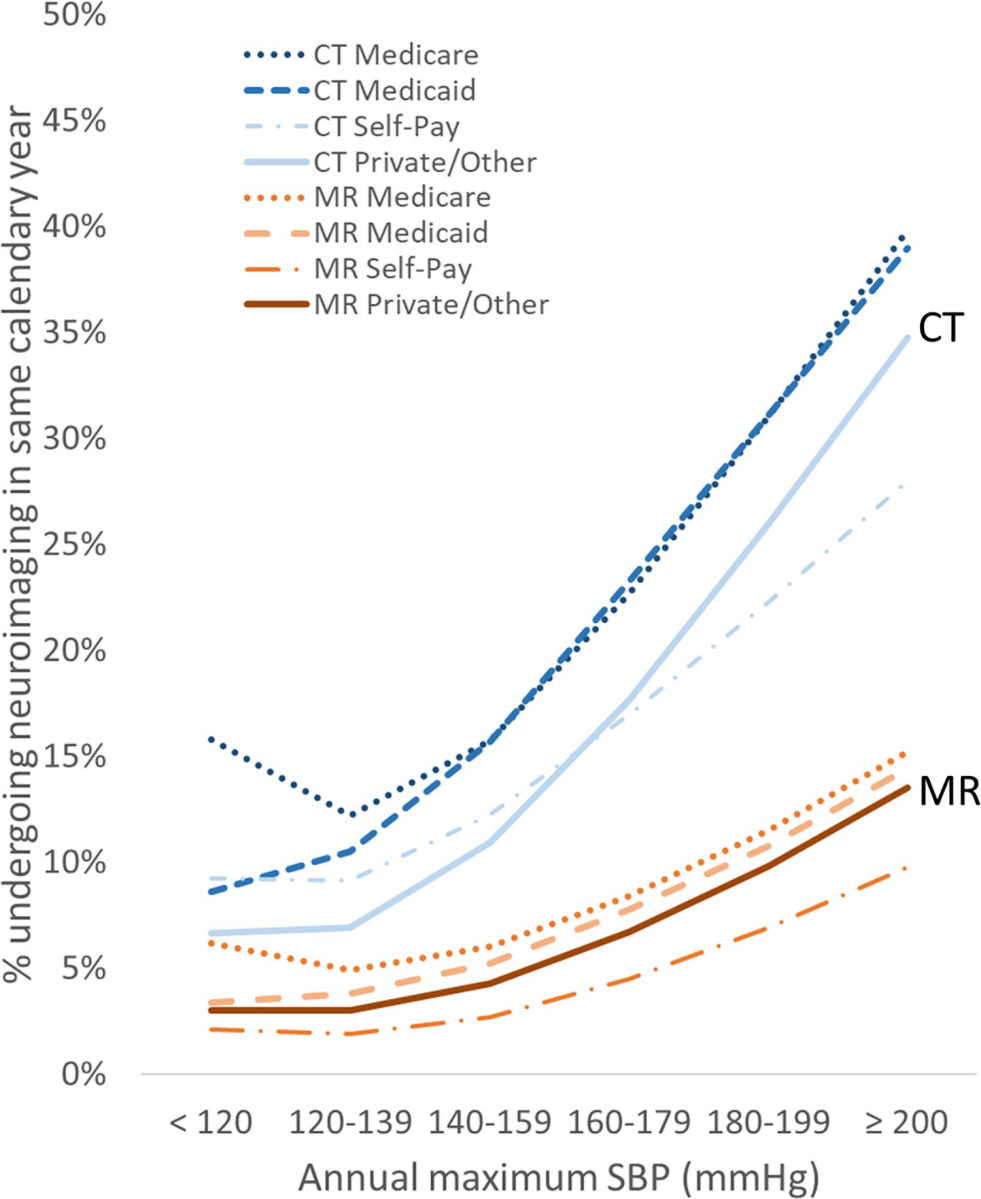
Same-year neuroimaging utilization stratified by payor type increases with increasing annual maximum SBP.

**Fig 8:**
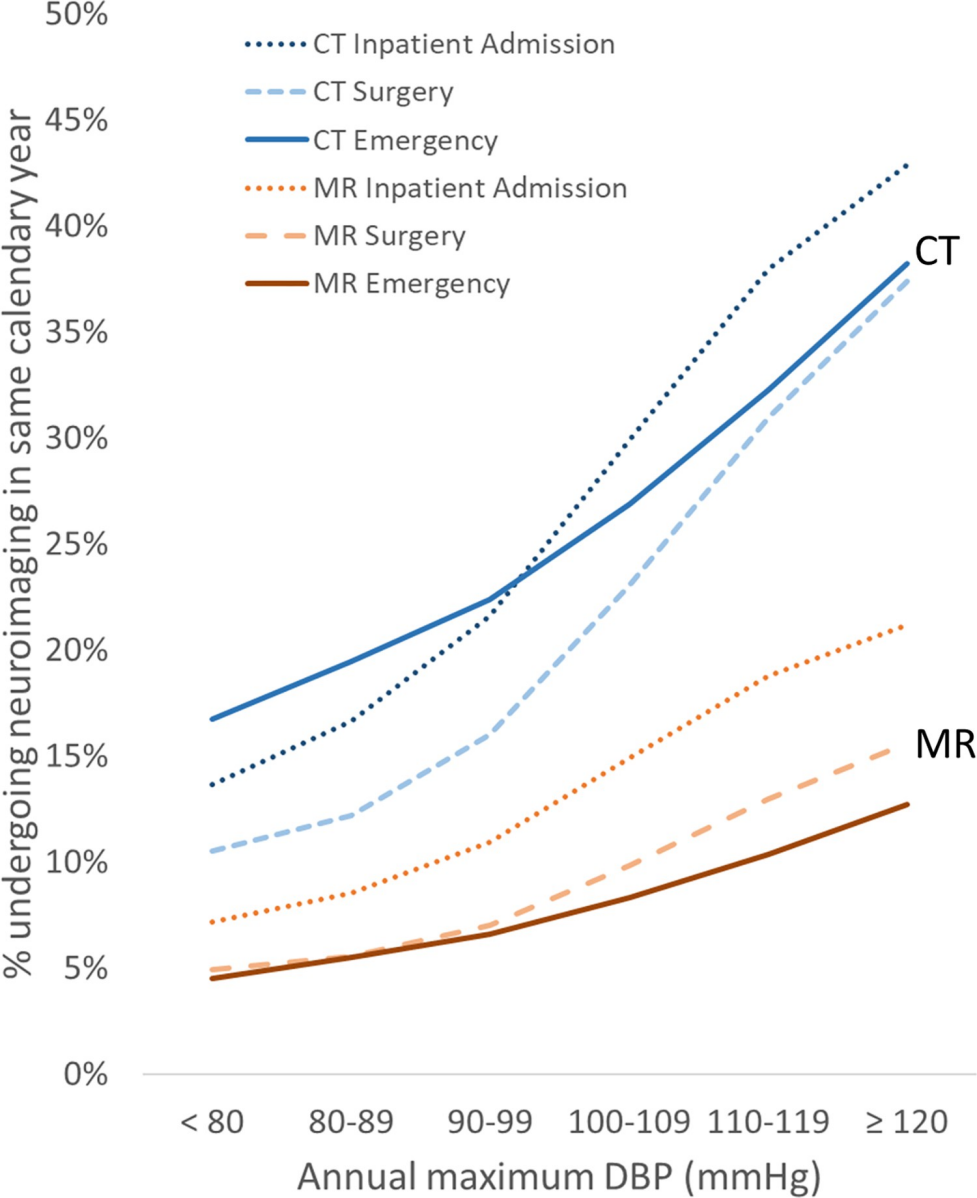
Same-year neuroimaging utilization stratified by encounter type increases with increasing annual maximum DBP.

**Fig 9:**
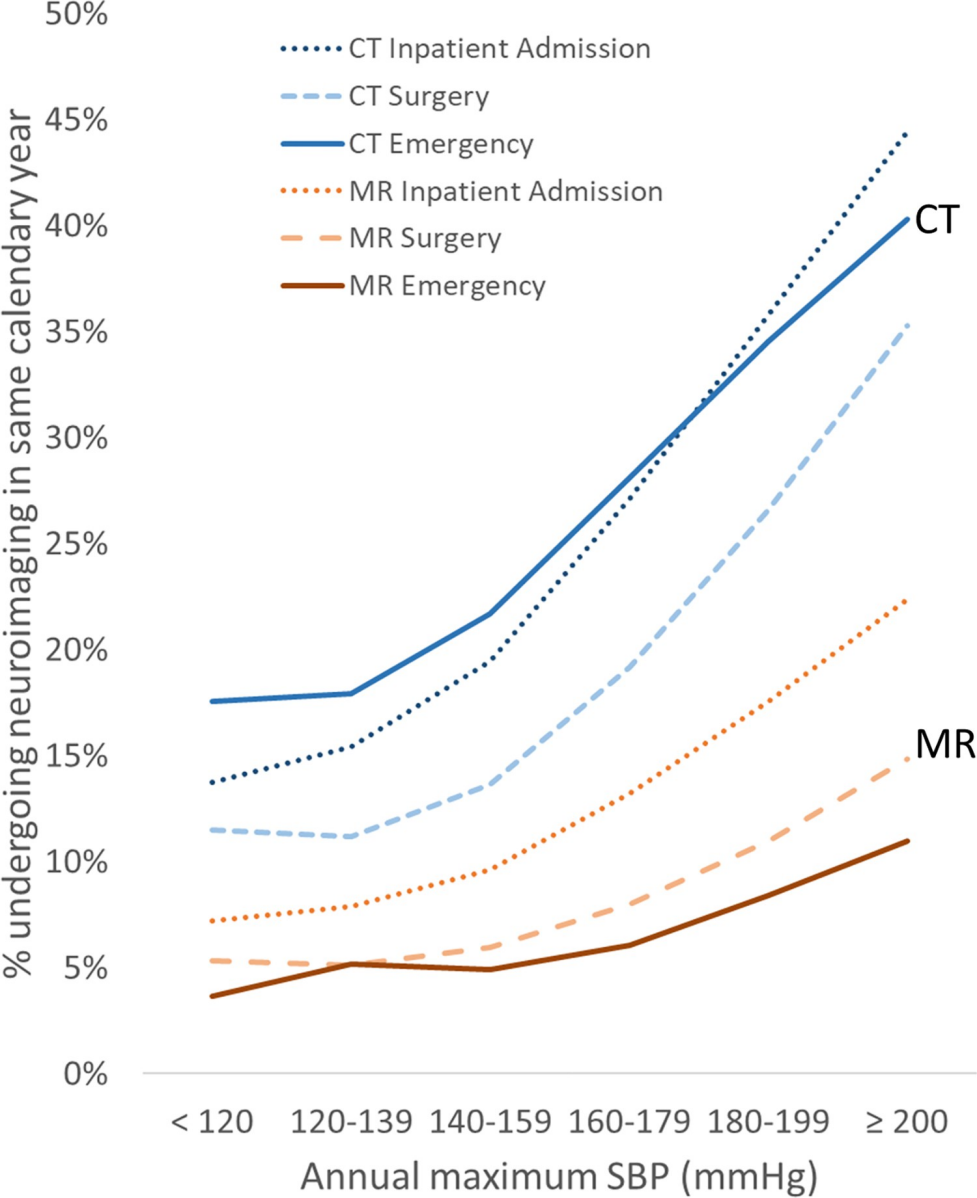
Same-year neuroimaging utilization stratified by encounter type increases with increasing annual maximum SBP.

Payor type showed relatively mild global effects on CT and MR imaging, with the Medicare group consistently showing higher utilization than the self-pay group (Figs 6 and 7). Within each payor type grouping, relative changes in utilization across BP appear relatively similar to the whole study population (Figs 1 and 2), except for the self-pay group, which shows a shallower slope of increased CT utilization at higher BP ranges compared to the other groups (Figs 6 and 7). The BP-utilization curves following stratifying by the encounter types of Emergency, Inpatient Admission, and Surgery are shown in Figs 8 and 9. In general, CT utilization is higher for inpatient and emergency encounters than for surgery encounters. For MR, neuroimaging rates are higher for inpatients and lowest for emergency encounters. Of note, the BP-utilization curve for CT among emergency encounters shows a mildly lower average slope than for other encounters, related to a slightly disproportionately higher frequency of imaging among low-BP groups in the emergency setting (Figs 8 and 9).

## Discussion

The current study demonstrates a strong quantitative relationship between annual maximum recorded blood pressure and the probability of undergoing at least one CT or MR neuroimaging study in the same year, with the effect more apparent among older individuals. The broad multi-institutional, national patient population used in this study shows a nonlinear dependence of utilization across the examined blood pressure ranges, with markedly higher rates of utilization among patients with higher blood pressures.

Previous studies have not investigated the quantitative relationship between hypertension and neuroimaging utilization. Since imaging exams in clinical practice are typically not performed for asymptomatic increases in blood pressure, the observed increase in neuroimaging utilization at higher maximum annual blood pressures may be explained by a combination of various mechanisms. For instance, uncontrolled hypertension is associated with increased incidence of correlated disease processes that may require imaging, such as increased incidence of stroke in untreated hypertension [13]. Other neurologic conditions, such as intracranial hemorrhage, hypertensive encephalopathy, pre-eclampsia/eclampsia, and dementia, are also associated with elevated BP [14]. A provider caring for a patient with poor BP control may be more likely to request head imaging if he or she perceives that the patient is at greater risk of a neurovascular complication or otherwise has elevated clinical suspicion for intracranial pathology, even though those imaged patients may not necessarily be eventually diagnosed with CVA or other at-risk diseases once evaluation is completed. Our observations on same-year CVA diagnosis and mortality across these cohorts defined by maximum annual BP (Table 2 and Fig 5) suggest that much of the higher neuroimaging utilization for the high maximum-BP cohorts is likely justified given their higher CVA and mortality risks. The current data suggest that age, which is associated with higher health care utilization in general [15], explains some but not all of the observed quantitative changes in utilization across BP levels, since a similar quantitative dependence persists after partitioning the cohort by patient age. However, we found age to influence the steepness of the utilization-BP curves, such that older patients with higher maximum annual BPs have much higher utilization than younger patients with similar BP ranges. A plausible explanation for this observation is the higher prevalence of various comorbidities with advanced age that contribute to a greater need for imaging, some of which may not be related to hypertension or cardiovascular risk. Payor status and encounter type affect utilization similarly for most BP groups, with a few exceptions, such as a mildly higher CT utilization for low-BP cohorts in the emergency setting and a mildly lower CT utilization for high-BP cohorts among self-payors. The higher utilization among Medicare beneficiaries has been previously reported and could be due to a confounding age effect described above and/or issues related to healthcare access/availability [16,17]. The relatively higher CT utilization among emergency encounters at lower BP ranges may be due to a lower general threshold of head CT imaging among emergency presentations. Prior studies had reported the rate of emergent CT and MR imaging, including neuroimaging, has grown over the past several decades [18].

Although the temporal change in utilization over time was not a primary objective of our study, we did observe mild increases in CT and MR utilization over the 5-year study period. Similar relative increases in head and spine MR imaging were reported for a non-U.S. population during the same time period [19]. Mild increases in CT and MR utilization in the U.S. had also been reported for the decades preceding our study period [20,21]. The apparent changes in utilization rates over time in our data should be interpreted with caution, as there are multiple potential variables affecting the rates reported in Table 1. For instance, capture rate of imaging exams may have increased over time based on growing numbers of participating institutions. Based on our currently available data, we are not able to definitively ascertain causality for the temporal change in utilization rates, and any causative association is likely multifactorial, with such factors as health care access, changes in other comorbidities, provider ordering trends, and numerous other variables likely contributing.

Compared to existing literature, the use of multi-institutional Cosmos data in our study offers several advantages related to its inclusion of diverse patient populations and practice settings. For instance, existing studies on Medicare data do not capture utilization from all segments of the population. In addition, studies performed on patients at academic medical centers may be biased toward more complex pathology or may include higher numbers of subspecialty referrals, whereas in the Cosmos platform, nonacademic health systems outnumber academic health systems by a factor of 2 [12]. Our approach illustrates the use of cross-institutional data aggregation to obtain population utilization rates that may be less biased than what might be obtained with single-institution or single-payor datasets.

This study offers several clinical implications. Although assessment of causality was not a goal of our study, the quantitative relationships by which BP and age correlate with neuroimaging utilization may facilitate subsequent efforts to quantify the impact of this modifiable risk factor on a major component of healthcare expenditures, namely medical imaging. We believe our results, which provide a holistic snapshot of population-level “all-cause” imaging, may be suitable for such purposes as assisting resource allocation for a given population or benchmarking cross-sectional imaging use across different subsets of the population to optimize healthcare expenditures. The use of blood pressure to define at-risk populations facilitates practical application because this metric is so widely measured and recorded during clinical encounters. Furthermore, the methodological approach used in this study may also be applied in future studies to assess other population-level risk factors across a broad multi-institutional patient population. This study also demonstrates the potential capability of big-data platforms to facilitate analysis of anonymized data that is periodically updated and aggregated from actual clinical patient records, permitting monitoring of health outcomes and risks for large patient cohorts with minimal data collection lag and at a relatively high frequency (approximately monthly based on the current refresh rate of the data). This study may also lay the groundwork for more sophisticated predictive modeling approaches to investigate contributions of various comorbidities and other risk factors on imaging utilization rates. Given our findings of a strong association between BP and imaging utilization, comprehensive hypertension management may be considered to lessen the growing strain on healthcare resources. While it is reasonable to suggest that focused efforts on blood pressure control may lead to less use of advanced imaging and consequently lower healthcare costs, it remains uncertain how much of the higher utilization rates among hypertensive patients can be mitigated by efforts to improve blood pressure control, and further studies are needed to prove effectiveness of any such intervention on utilization and/or costs.

One strength of our study design is the use of a vendor-facilitated aggregation platform to obtain data from a large multi-institutional population. This compensates for any limitations related to overestimation of utilization when reporting single-institution data from a tertiary referral academic medical center, in which higher-complexity or higher-acuity patients may be overrepresented. The use of aggregate data does have limitations, including the inability to investigate effects of a larger number and granularity of data variables or apply multivariable statistical or computational approaches to generate robust prediction models. This limitation represents a methodological trade-off for the Cosmos data platform in that the ability to access this large multi-institutional patient population is offset by limitations in our ability to access individual patient-level records for more sophisticated predictive modeling, such as logistic regression. As a result, quantitative effects of multiple potential confounding variables, such as various comorbidities, were not specifically investigated in this study. Likelihood of imaging and prevalence of neurovascular disease may be affected my numerous variables not examined in this study, such as coexisting diabetes mellitus, hyperlipidemia, atrial fibrillation, coronary artery disease, family history of stroke, smoking history, previous history of cerebral vascular disease, and physical exam findings such as carotid bruits. While the current study presents a quantitative assessment of an empiric relationship between BP values and neuroimaging utilization across a population, potential contributory effects of other clinical data variables on utilization were not specifically analyzed in this study. Data completeness and differences in encoding or capturing of imaging exams of interest across institutions may represent a potential concern when analyzing multi-institutional data, but various mechanisms are in place within the Cosmos computational framework to standardize data variables across institutions [12].

In summary, there is a strong positive relationship between CT and MR neuroimaging utilization rates and documented maximum annual blood pressures. The study quantifies the effect of hypertension, particularly poorly controlled hypertension, across all age groups and demonstrates greater effects among older patients. These observations may help inform public health efforts on hypertension management as a potential approach to mitigate growing imaging utilization and associated costs, although additional studies are needed to establish effectiveness of any preventive intervention.

## Supporting information

S1 File (XLSX)

## References

[1] [Anonymous]. Hypertension. cdc.gov. 2019. Available: https://www.cdc.gov/nchs/fastats/hypertension.htm.

[2] Cushman WC, Ford CE, Cutler JA, Margolis KL, Davis BR, Grimm RH, et al. Blood pressure control in the anthihypertensive and lipid lowering treatment to prevent heart attack trial (ALLHAT). American Journal of Hypertension. 1998;11. doi: 10.1016/s0895-7061(97)90762-7 8722437

[3] Paramore LC, Halpern MT, Lapuerta P, Hurley JS, Frost FJ, Fairchild DG, et al. Impact of poorly controlled hypertension on healthcare resource utilization and cost. The American journal of managed care. 2001;7: 389–398. 11310193

[4] Kelly DM, Rothwell PM. Blood pressure and the brain: the neurology of hypertension. Practical neurology. 2020;20: 100–108. doi: 10.1136/practneurol-2019-002269 31558584

[5] Wajngarten M, Silva GS. Hypertension and Stroke: Update on Treatment. Eur Cardiol. 2019;14: 111. doi: 10.15420/ecr.2019.11.1 31360232 PMC6659031

[6] Lewington Sarah, Clarke Robert, Qizilbash Nawab, Peto Richard, Collins Rory. 1-s2.0-S0140673602119118-main. 2002. 10.1016/s0140-6736(02)11911-8 12493255

[7] Weaver Colin G., Clement Fiona M., Norm R.C Campbell, Matthew T. James, Scott W Klarenbach, Brenda R. Hemmelgarn, Marcello Tonelli, Kerry A. McBrien, and for the Alberta Kidney Disease Network and the Interdisciplinary Chronic Disease Collaboration. HYPERTENSIONAHA.115.05702 (1). Volume 66, Issue 3, Pages 502–508. 2015.

[8] Forouzanfar MH, Liu P, Roth GA, Ng M, Biryukov S, Marczak L, et al. Global Burden of Hypertension and Systolic Blood Pressure of at Least 110 to 115 mm Hg, 1990–2015. JAMA: the journal of the American Medical Association. 2017;317: 165–182. doi: 10.1001/jama.2016.19043 28097354

[9] Burke JF, Skolarus LE, Callaghan BC, Kerber KA. Choosing Wisely: Highest-cost tests in outpatient neurology. Ann Neurol. 2013;73: 679–683. https://doi-org.proxy.lib.ohio-state.edu/10.1002/ana.23865. 23595536 10.1002/ana.23865 PMC5570467

[10] Bernardy M MD, Ullrich CG MD, Rawson JV MD, Allen B MD, Thrall JH MD, Keysor KJ BS, et al. Strategies for Managing Imaging Utilization. Journal of the American College of Radiology. 2009;6: 844–850. doi: 10.1016/j.jacr.2009.08.003 19945039

[11] Wong HJ, Sistrom CL, Benzer TI, Halpern EF, Morra DJ, Gazelle GS, et al. Use of Imaging in the Emergency Department: Physicians Have Limited Effect on Variation. Radiology. 2013;268: 779–789. doi: 10.1148/radiol.13130972 23801769

[12] Tarabichi Y, Frees A, Honeywell S, Huang C, Naidech AM, Moore JH, et al. The Cosmos Collaborative: A Vendor-Facilitated Electronic Health Record Data Aggregation Platform. ACI open. 2021;05: e36. doi: 10.1055/s-0041-1731004 35071993 PMC8775787

[13] XinfangXie MD, EmilyAtkins BHlthSc, ProfJicheng Lv MD Alexander Bennett BMedSc, Prof Bruce Neal MBChB Prof Toshiharu Ninomiya PhD, Prof Mark Woodward PhD, Prof Stephen MacMahon PhD, Fiona Turnbull PhD, Prof Graham S Hillis MBChB, Prof John ChalmersMBBS, Prof Jonathan Mant MD, AbdulSalam MPharm, Prof Kazem RahimiPhD, Prof Vlado Perkovic MBBS, Prof Anthony Rodgers MBChBb. Effects of intensive blood pressure lowering on cardiovascular and renal outcomes:updated systematic review and meta-analysis. Science Direct. 2016;387: 435–443. 10.1016/S0140-6736(15)00805-3 26559744

[14] Kelly DM, Rothwell PM. Blood pressure and the brain: the neurology of hypertension. Practical neurology. 2020;20: 100–108. doi: 10.1136/practneurol-2019-002269 31558584

[15] Kalseth J, Halvorsen T. Health and care service utilisation and cost over the life-span: a descriptive analysis of population data. BMC health services research. 2020;20: 435. doi: 10.1186/s12913-020-05295-2 32429985 PMC7236310

[16] Burke JF, Kerr EA, McCammon RJ, Holleman R, Langa KM, Callaghan BC. Neuroimaging overuse is more common in Medicare compared with the VA. Neurology. 2016;87: 792–798. doi: 10.1212/WNL.0000000000002963 27402889 PMC4999324

[17] Wang JJ, Pelzl CE, Boltyenkov A, Katz JM, Hemingway J, Christensen EW, et al. Updated Trends, Disparities, and Clinical Impact of Neuroimaging Utilization in Ischemic Stroke in the Medicare Population: 2012 to 2019. J Am Coll Radiol. 2022;19: 854–865. doi: 10.1016/j.jacr.2022.03.008 35483436 PMC9308737

[18] Prabhakar AM, Gottumukkala RV, Hemingway J, Hughes DR, Patel SS, Duszak RJ. Increasing utilization of emergency department neuroimaging in Medicare beneficiaries from 1994 to 2015. Am J Emerg Med. 2018;36: 680–683. doi: 10.1016/j.ajem.2017.12.057 29306644

[19] Luxenburg O, Saban M, Myers V, Vaknin S, Boldor N, Wilf-Miron R. National and regional trends in MRI utilization in the face of the ongoing COVID-19 pandemic. Isr J Health Policy Res. 2021;10: 40-y. doi: 10.1186/s13584-021-00472-y 34266476 PMC8280577

[20] Smith-Bindman R, Kwan ML, Marlow EC, Theis MK, Bolch W, Cheng SY, et al. Trends in Use of Medical Imaging in US Health Care Systems and in Ontario, Canada, 2000–2016. JAMA. 2019;322: 843–856. doi: 10.1001/jama.2019.11456 31479136 PMC6724186

[21] Hoang JK, Choudhury KR, Eastwood JD, Esclamado RM, Lyman GH, Shattuck TM, et al. An exponential growth in incidence of thyroid cancer: trends and impact of CT imaging. AJNR Am J Neuroradiol. 2014;35: 778–783. doi: 10.3174/ajnr.A3743 24113469 PMC7965799

